# Structural, Mechanical, Anisotropic, and Thermal Properties of AlAs in *o*C12 and *h*P6 Phases under Pressure

**DOI:** 10.3390/ma11050740

**Published:** 2018-05-07

**Authors:** Wei Zhang, Changchun Chai, Yanxing Song, Qingyang Fan, Yintang Yang

**Affiliations:** Key Laboratory of Ministry of Education for Wide Band-Gap Semiconductor Materials and Devices, School of Microelectronics, Xidian University, Xi’an 710071, China; zw_xidian@163.com (W.Z.); ccchai@mail.xidian.edu.cn (C.C.); syx739686768@163.com (Y.S.); ytyang@xidian.edu.cn (Y.Y.)

**Keywords:** *o*C12 phase-AlAs, *h*P6 phase-AlAs, mechanical properties, anisotropic properties, thermal properties

## Abstract

The structural, mechanical, anisotropic, and thermal properties of *o*C12-AlAs and *h*P6-AlAs under pressure have been investigated by employing first-principles calculations based on density functional theory. The elastic constants, bulk modulus, shear modulus, Young’s modulus, *B*/*G* ratio, and Poisson’s ratio for *o*C12-AlAs and *h*P6-AlAs have been systematically investigated. The results show that *o*C12-AlAs and *h*P6-AlAs are mechanically stable within the considered pressure. Through the study of lattice constants (*a*, *b*, and *c*) with pressure, we find that the incompressibility of *o*C12-AlAs and *h*P6-AlAs is the largest along the *c*-axis. At 0 GPa, the bulk modulus *B* of *o*C12-AlAs, *h*P6-AlAs, and diamond-AlAs are 76 GPa, 75 GPa, and 74 Gpa, respectively, indicating that *o*C12-AlAs and *h*P6-AlAs have a better capability of resistance to volume than diamond-AlAs. The pressure of transition from brittleness to ductility for oC12-AlAs and hP6-AlAs are 1.21 GPa and 2.11 GPa, respectively. The anisotropy of Young’s modulus shows that *o*C12-AlAs and *h*P6-AlAs have greater isotropy than diamond-AlAs. To obtain the thermodynamic properties of *o*C12-AlAs and *h*P6-AlAs, the sound velocities, Debye temperature, and minimum thermal conductivity at considered pressure were investigated systematically. At ambient pressure, *o*C12-AlAs (463 K) and *h*P6-AlAs (471 K) have a higher Debye temperature than diamond-AlAs (433 K). At *T* = 300 K, *h*P6-AlAs (0.822 W/cm·K^−1^) has the best thermal conductivity of the three phases, and *o*C12-AlAs (0.809 W/cm·K^−1^) is much close to diamond-AlAs (0.813 W/cm·K^−1^).

## 1. Introduction

Group III–V compound semiconductor materials are the “core” of solid-state light sources and power electronic devices because of their large band gap, high breakdown field, high thermal conductivity, high saturated electron drift velocity, strong radiation resistance, and superior performance [1,2,3,4,5,6,7]. They have broad application prospects in semiconductor lighting, new generation mobile communications, energy Internet, high-speed rail transportation, new energy vehicles, consumer electronics, and other fields, and it is hoped that these materials will break through the bottleneck of traditional semiconductor technology [8,9,10,11,12]. Among these compound semiconductors, GaN, AlN, AlP, and AlAs have been of considerable interest, because understanding their structural and electronic properties is crucial to semiconductor technological applications. First-principles calculations based on density functional theory (DFT) represent one of the most accurate microscopic theories in materials science. Advances in the accuracy and efficiency of first-principles electronic structure calculations play an increasingly important role in the prediction of material structures and properties.

In ref. [13], Mujica et al. provided a detailed review of the current known structures, high-pressure behavior, and theoretical work on the group III–V compound semiconductor materials. Under normal conditions, AlN and GaN crystallize in the wurtzite structure, and they may also form a zinc blende structure when the epitaxial growth technique is used [14]. The first-principles calculations confirm that the zinc blende structure is metastable, although it can lie close in enthalpy (<50 meV) to that of the stable wurtzite structure [15]. For orthorhombic GaN (*Pnma*-GaN), it was evaluated that GaN will have a direct band gap of 1.85 eV, and that *Pnma*-GaN is mechanically and dynamically stable at ambient pressure [16]. Using the CALYPSO (Crystal structure AnaLYsis by Particle Swarm Optimization) code, Liu et al. [17] investigated four novel AlN phases (*Pmn2*_1_-AlN, *Pbam*-AlN, *Pbca*-AlN, and *Cmcm*-AlN), and proved that these four novel AlN phases are more favorable in thermodynamics than the rock-salt structure at ambient pressure, and can be transformed to the rock-salt structure under certain pressures. Using first-principles calculations, the four predicted novel AlN phases, which are wide direct band-gap semiconductors with band gaps of 5.95 (*Pmn*2_1_-AlN), 5.99 (*Pbam*-AlN), 5.88 (*Pbca*-AlN), and 5.59 eV (*Cmcm*-AlN), were calculated in detail [18]. The phase stability, mechanical, and optoelectronic properties of *bct*-AlN (at ambient pressure) and *h*-AlN (at higher pressure) were investigated by Yang et al. [19]. Their investigation proved that *bct*-AlN is mechanically and dynamically stable at ambient pressure, that the *h*-AlN phase can be stabilized by increasing pressure, and that it is mechanically and dynamically stable at 10 GPa. For the AlP semiconductors with four novel AlN phases (*Pmn*2_1_-AlP, *Pbam*-AlP, *Pbca*-AlP, and *bct*-AlP), the electronic properties are calculated by a hybrid functional [20]. All four of these novel AlN phases behave in a ductile manner, and the band gaps are 3.22 eV, 3.27 eV, 3.47 eV, and 3.04 eV for *Pmn2*_1_-AlP, *Pbam*-AlP, *Pbca*-AlP, and *bct*-AlP, respectively. In 1983, Froyen and Cohen [21] investigated the static and structural properties of AlAs. In 1994, Greene et al. [22] studied the crystal structure of AlAs in a diamond anvil cell using energy-dispersive X-ray diffraction to 46 GPa; this study was the first experimental observation of AlAs transforming from the zinc blende structure to the NiAs structure, and the equilibrium transformation pressure was reported to be 7 ± 5 GPa. Liu et al. [23] proved that AlAs can transition from the zinc blende structure to the NiAs structure at 6.1 GPa, which is in agreement with Greene’s work. Mujica et al. [24] studied the phase stability of AlAs, including zinc blende, wurtzite, NaCl, CsCl, *β*-tin, NiAs, and *sc*16 structures, and proved that *sc*16-AlAs is not thermodynamically stable at any pressure, whereas CsCl-AlAs is thermodynamically stable at very high pressures. An ab initio total energy investigation of the high-pressure phase diagrams (including *Cmcm* and cinnabar) of AlAs was conducted by Mujica et al. [25] to prove that the *Cmcm* structure is stable within a certain pressure range, and that the cinnabar structures are not thermodynamically stable at any pressure. Srivastava et al. [26] studied the stability of the AlAs using the local density approximation and the generalized gradient approximation potential. Their study revealed that under the application of pressure, the zinc-blende structure first transforms to the wurtzite structure at 3.88 GPa.

Utilizing first-principles calculations, the theoretical and experimental research studies of AlAs in diamond, zinc-blende, NiAs, rock-salt, wurtzite, NaC1, CsCl, *β*-tin, NiAs, wurtzite, and *sc*16 structures have been performed [21,22,23,24,25,26]. Recently, Liu et al. [27] investigated the phase transformation and properties of three metastable phases of AlAs (*h*P6-, *o*C12-, and *c*I24-AlAs). The detailed physical properties of *o*C12-AlAs and *h*P6-AlAs with the change in pressure have not yet been determined. Therefore, this work presents the structural, mechanical, anisotropic, and thermal properties of *o*C12-AlAs and *h*P6-AlAs under pressure.

## 2. Materials and Methods

First-principles calculations, which were applied to the theoretical investigations on AlAs in *o*C12 and *h*P6 phases, were performed using density functional theory (DFT) [28,29] based on the Cambridge Series Total Energy Package (CASTEP) code [30]. All of the calculations were performed with the generalized gradient approximation (GGA) in the form of the Perdew–Burke–Ernzerhof (PBE) functional [31,32,33] for the exchange correlation potential. The Al-3*s*^2^3*p* and As-4*s*^2^4*p*^3^ were regarded as the valence electron structures. To ensure the precision at 1 meV, the plane-wave pseudopotential method was employed; the energy cut-off *E*_cut_ was 550 eV, and the *k*-point sampling of the Brillouin zones constructed using the Monkhorst–Pack scheme were 11 × 11 × 4, 6 × 10 × 4 grids for AlAs in *h*P6 and *o*C12 phases in a conventional cell. The Broyden–Fletcher–Goldfarb–Shanno (BFGS) [34] minimization was applied to the geometry optimization, and the thresholds of the converged structures were as follows: the total energy tolerance was 5 × 10^−6^ eV/atom; the maximum force on the atom was 0.01 eV/Å; the maximum ionic displacement was less than 5 × 10^−4^ Å; and the maximum stress was less than 0.02 GPa. The ultrasoft quasipotential method was used to describe the presence of tightly bound core electrons. In addition, the Voigt–Reuss–Hill approximation was employed to estimate the bulk modulus, shear modulus, and Young’s modulus. To obtain the elastic constants under various pressures, we consider the strains to be non-volume-conserving, because this method is consistent with our calculated elastic constants using the stress–strain coefficients.

## 3. Results and Discussion

### 3.1. Structural Properties

**Figure 1:**
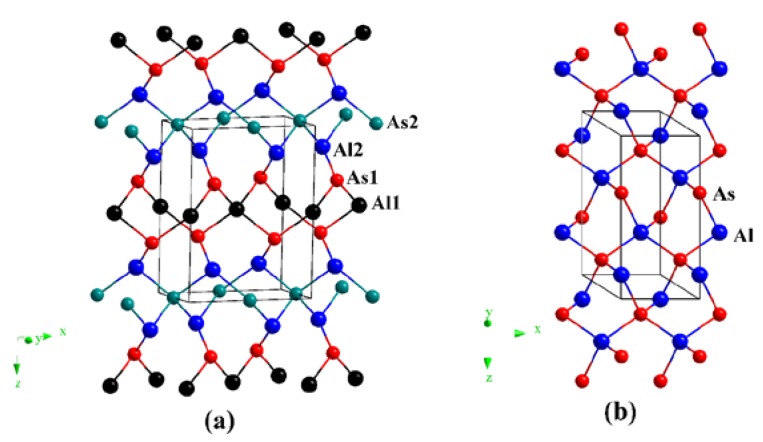
The crystal structures of AlAs in the *o*C12 phase (**a**) and *h*P6 phase (**b**).

**Figure 2:**
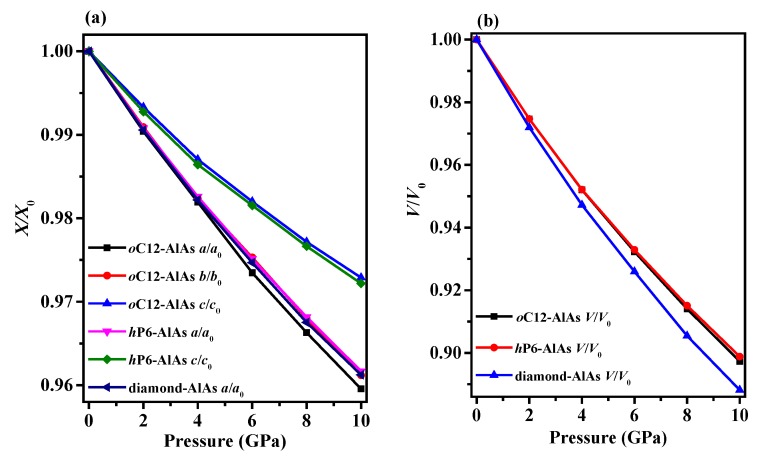
The lattice constants *X*/*X*_0_ compression (**a**) and primitive cell volume *V*/*V*_0_ (**b**) as functions of pressure for *o*C12-AlAs, *h*P6-AlAs, and diamond-AlAs.

The crystal structures of *o*C12-AlAs and *h*P6-AlAs are shown in Figure 1. They were obtained with the lowest-energy structure at the same stoichiometry. The *o*C12-AlAs is a C-centered orthorhombic crystal system (space group *C*222) with 12 atoms per unit cell, including four Al atoms and four As atoms. At zero pressure, within the structure of *o*C12, four inequivalent atoms represented as Al1, Al2, As1, and As2 occupy the crystallographic 2*d*, 4*k*, 4*k*, and 2*b* sites in the conventional cell, respectively, which are 2*d* sites (0.00000, 0.00000, 0.50000), 4*k* sites (0.25000, 0.25000, 0.16735), 4*k* sites (0.25000, 0.25000, 0.66468) and 2*b* sites (0.50000, 0.00000, 0.00000). The *h*P6-AlAs is a primitive centered hexagonal structure (space group *P*6_4_22) with six atoms per unit cell, including three Al atoms and three As atoms. At zero pressure, regarding the atomic positions of *o*C12-AlAs, Al atoms occupy the crystallographic 3*c* sites (0.50000, 0.00000, 1.00000), and As atoms occupy the crystallographic 3*d* sites (0.50000, 0.00000, 0.50000). All of the atoms in *o*C12-AlAs and *h*P6-AlAs combine to form Al–As bonds, indicating that no Al–Al (As–As) bonds were presented in the *o*C12 and *h*P6 structures. The equilibrium crystal lattice parameters of *o*C12-AlAs and *h*P6-AlAs at zero pressure are listed in Table 1; in addition, the optimized lattice parameters and experimental values for diamond-AlAs at zero pressure are also listed in Table 1. In this work, the lattice parameter of diamond-AlAs is 5.675 Å, which is consistent with the experimental value 5.661 Å [35], indicating that our results are valid and realistic. For *o*C12-AlAs, the lattice constants are *a* = 6.972 Å, *b* = 3.968 Å, and *c* = 9.108 Å; for *h*P6-AlAs, the lattice constants are *a* = *b* = 4.019 Å and *c* = 8.990 Å. For *o*C12-AlAs and *h*P6-AlAs, the lattice parameters *a*, *b,* and *c* in this work are also clearly consistent with those in the previous report [27], providing further evidence of the accuracy of our work. To compare the incompressibility of *o*C12-AlAs, *h*P6-AlAs, and diamond-AlAs under pressure, the lattice constants *X*/*X*_0_ compression and primitive cell volume *V*/*V*_0_ as functions of pressure are shown in Figure 2. As shown in Figure 2, both the lattice constants’ *X*/*X*_0_ compression and the primitive cell volume *V*/*V*_0_ have negative slopes, illustrating that as the pressure increases, decreases occur in the lattice constants and the primitive cell volume. In Figure 2a, for *o*C12-AlAs, the incompressibility along the *b*-axis is less than that along the *c*-axis, but it is larger than that along the *a*-axis. For *h*P6-AlAs, the incompressibility along the *c*-axis is larger than that along the *a*-axis (*b*-axis). The lattice constants’ ratios *X*/*X*_0_ clearly indicate the elastic anisotropy of both *o*C12-AlAs and *h*P6-AlAs. Along the *a*-axis, the incompressibility of diamond-AlAs is slightly less than that of *h*P6-AlAs, but it is larger than that of *o*C12-AlAs. Along the *b*-axis, the incompressibility of *h*P6-AlAs and *o*C12-AlAs are almost equal and both are larger than that of diamond-AlAs. Along the *c*-axis, the incompressibility of *h*P6-AlAs is less than that of *o*C12-AlAs, but it is larger than that of diamond-AlAs. From Figure 2b, we can see that the volume incompressibility is similar to that of *a*/*a*_0_, *b*/*b*_0_, and *c*/*c*_0_. The volume compressibility of *o*C12-AlAs is slightly larger than that of *h*P6-AlAs, but less than that of diamond-AlAs.

### 3.2. Mechanical Properties

The elastic constants of *o*C12-AlAs, *h*P6-AlAs, and diamond-AlAs at different pressures are listed in Table 2, which are used to analyze the mechanical stability. At zero pressure, the elastic constants of *o*C12-AlAs and *h*P6-AlAs in this work are in excellent agreement with the results of previous research [27], and the elastic constants of diamond-AlAs are also clearly consistent with the available experimental data [36], proving that our work is accurate and trustworthy. The orthorhombic phase has nine independent elastic constants (*C*_11_, *C*_12_, *C*_13_, *C*_22_, *C*_23_, *C*_33_, *C*_44_, *C*_55_, and *C*_66_). The mechanical stability criteria of the orthorhombic structure are shown below [37]:

(1) $$C_{ii}>0,i=1,2,3,4,5,6,$$

(2) $$[C_{11}+C_{22}+C_{33}+2(C_{12}+C_{13}+C_{23})]>0,$$

(3) $$(C_{11}+C_{22}-2C_{12})>0,$$

(4) $$(C_{11}+C_{33}-2C_{13})>0,$$

(5) $$(C_{22}+C_{33}-2C_{23})>0,$$

The hexagonal structure has five independent elastic constants (*C*_11_, *C*_12_, *C*_13_, *C*_33_, and *C*_44_,). The mechanical stability criteria of the hexagonal phase are shown below [38]:

(6) $$C_{44}>0,$$

(7) $$C_{11}>\left|C_{12}\right|,$$

(8) $$(C_{11}+2C_{12})C_{33}>2C_{13}^{2},$$

According to the abovementioned criteria, all of the independent elastic constants of *o*C12-AlAs and *h*P6-AlAs at different pressures are positive and satisfy the mechanical stability criteria, indicating that *o*C12-AlAs and *h*P6-AlAs are mechanically stable under the considered pressure. The elastic constants *C*_11_, *C*_12_, *C*_13_, *C*_22_, *C*_23_, and *C*_33_ increase with different rates under increasing pressure, whereas there is no apparent regular pattern in the changes of *C*_44_, *C*_55_, and *C*_66_. The elastic constants *C*_11_, *C*_22_, and *C*_33_ denote the resistance to linear compression along the *a*, *b*, and *c* axes, respectively. For example, *o*C12-AlAs has a larger *C*_22_ than *C*_11_, but it is smaller than *C*_33_, which manifested that its *b*-axis is less compressible than its *a*-axis, but more compressible than its *c*-axis. At ambient pressure, both *o*C12-AlAs and *h*P6-AlAs have larger *C*_11_, *C*_22_, and *C*_33_ than *C*_11_ of diamond-AlAs, which manifested that *o*C12-AlAs and *h*P6-AlAs have a stronger resistance to linear compression than diamond-AlAs. These results are consistent with the conclusions in the preceding part of this paper. In addition, all of the above three elastic constants (*C*_11_, *C*_22_, and *C*_33_) increase under increasing pressure, indicating that the greater the pressure, the more favorable the mechanical properties of *o*C12-AlAs and *h*P6-AlAs along the *a*, *b*, and *c* axes will be.

The elastic modulus, including bulk modulus *B*, shear modulus *G*, Young’s modulus *E*, and Poisson’s ratio *v* of *o*C12-AlAs and *h*P6-AlAs are listed in Table 2. The larger the values of *B* and *G*, the better the capability of resistance to volume and shape change. The Young’s modulus *E* and Poisson’s ratio *v* are obtained by the following expressions [39,40,41]: *E* = 9*BG*/(3*B* + *G*) and *v* = (3*B* − 2*G*)/[2(3*B* + *G*)]. The results of *o*C12-AlAs, *h*P6-AlAs, and diamond-AlAs at zero pressure are consistent with the values in references [27,36], which show that the results are reliable. The Young’s modulus can be applied to describe the corresponding tensile strain. The higher the value of *E*, the stiffer the materials, and the Poisson’s ratio can indicate the stability of a crystal against shear deformation. A larger Poisson ratio means better plasticity. As listed in Table 2, the values of bulk modulus *B* and Poisson’s ratio *v* for *o*C12-AlAs and *h*P6-AlAs increased at different rates under increasing pressure. At zero pressure, the bulk modulus *B* of *o*C12-AlAs, *h*P6-AlAs, and diamond-AlAs is 76 GPa, 75 GPa, and 74 GPa, respectively, which indicated that *o*C12-AlAs’s capability of resistance to volume change is the best, and diamond-AlAs’s is the weakest. Also, the shear modulus *G* of *o*C12-AlAs, *h*P6-AlAs, and diamond-AlAs (at zero pressure) is 44 GPa, 46 GPa, and 44 GPa, respectively, which shows that the capability of resistance to shape change of *o*C12-AlAs and diamond-AlAs are almost equal, and both are worse than that of *h*P6-AlAs. The *o*C12-AlAs and *h*P6-AlAs have close numerical values, both in bulk modulus and shear modulus, leading to *o*C12-AlAs and *h*P6-AlAs having similar values for both Young’s modulus and Poisson’s ratio. The maximum values of shear modulus *G* and Young’s modulus *E* (48 GPa and 124 GPa, respectively) for *o*C12-AlAs are at 6 GPa; the maximum values of shear modulus *G* and Young’s modulus *E* (51 GPa and 131 GPa, respectively) for *o*C12-AlAs are also at 6 GPa. All of the values of bulk modulus *B* and *G* of *h*P6-AlAs at high pressures (≥6 GPa) are larger than those of *o*C12-AlAs, proving that *h*P6-AlAs have a larger capability of resistance to volume and shape change than *o*C12-AlAs.

**Figure 3:**
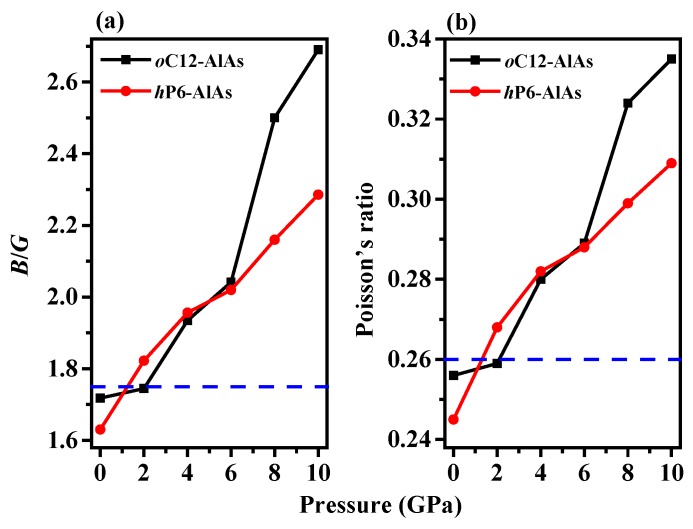
The ratio of bulk to shear modulus (*B*/*G*) (**a**) and Poisson’s ratio (**b**) of *o*C12-AlAs, *h*P6-AlAs, and diamond-AlAs as a function of pressure.

As proposed by Pugh, the brittle and ductile behavior of materials can be predicted by the ratio of bulk to shear modulus (*B*/*G*) [42]. The modulus ratio of *B*/*G* and value of *v* as functions of pressure are shown in Figure 3. The modulus ratio of *B*/*G* is an indication of the extent of the plastic range for a pure metal, with a high value of *B*/*G* (*B*/*G* > 1.75) being associated with malleability and a low value (*B*/*G* < 1.75) being associated with brittleness. In addition, the modulus ratio *B*/*G* is related to Poisson’s ratio *v* [42]: *B*/*G* = 2(1 + *v*)/[3(1 − 2*v*)] or *v* = (3*B*/*G* − 2)/(6*B*/*G* + 2). Therefore, Poisson’s ratio can also be used to quantify the malleability. A solid with a larger value of *ν* (*v* > 0.26) is ductile, whereas a solid with a lower value of *ν* is brittle. As shown in Figure 3, the modulus ratio *B*/*G* and Poisson’s ratio increase with pressure for both of the phases, indicating that the greater the pressure, the more favorable the ductile properties of *o*C12-AlAs and *h*P6-AlAs will be. The pressure of transition from brittleness to ductility are 1.21 GPa and 2.11 GPa for *o*C12-AlAs and *h*P6-AlAs, respectively.

### 3.3. Anisotropic Properties

**Figure 4:**
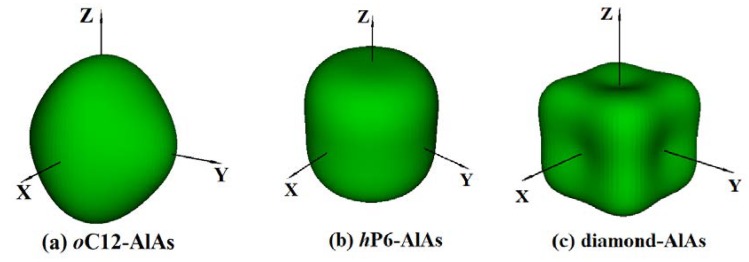
The three-dimensional (3D) surface constructions of Young’s modulus *E* for *o*C12-AlAs, *h*P6-AlAs, and diamond-AlAs at ambient pressure.

It is well-known that the anisotropy of elasticity is an important implication in engineering science and crystal physics. The three-dimensional (3D) surface construction is a valid method to describe the elastic anisotropy of a solid perfectly. For isotropic systems, the three-dimensional direction dependence will exhibit spherical symmetry, i.e., the physical, chemical, and other aspects of the nature of the materials will not change for different directions; the higher the spherical deviation, the higher the anisotropy content [43]. The 3D surface constructions of Young’s modulus *E* for *o*C12-AlAs, *h*P6-AlAs, and diamond-AlAs at zero pressure are shown in Figure 4 using ELAM codes (Elastic Anisotropy Measures) [44]. Obviously, the 3D surface constructions of the directional dependences of reciprocals of the Young’s modulus for the two novel materials are different because of their different crystal structures; these differences indicated that the Young’s modulus for these three phases show some mechanical anisotropy. Regarding the orthorhombic structure *o*C12 phase (Figure 4a), the 3D surface constructions of Young’s modulus along the *x*, *y*, and *z*-axis deviate from the spherical shape largely, i.e., the *o*C12-AlAs has high anisotropy in Young’s modulus. The 3D surface constructions of the Young’s modulus for the hexagonal structure *h*P6 phase (Figure 4b) has a smaller amount of deviation than that of the *o*C12 phase from the sphere, indicating that the Young’s modulus for the *h*P6 phase shows a larger isotropy than that of the *o*C12-AlAs. The maximum value (minimum value) of *o*C12-AlAs, *h*P6-AlAs, and diamond-AlAs is 121 GPa (100 GPa), 124 GPa (104 GPa), and 132 GPa (83 GPa), respectively. The *E*_max_/*E*_min (*o*C12-AlAs)_ = 1.21, *E*_max_/*E*_min (*h*P6-AlAs)_ = 1.19, and *E*_max_/*E*_min (diamond-AlAs)_ = 1.59 show that diamond-AlAs has the greatest anisotropy, and *h*P6-AlAs has the least anisotropy of the three structures.

Following the procedure of Brugger [45], the single-crystal elastic constants can be applied to calculate the phase velocities of pure transverse and longitudinal modes; these phase velocities can indicate the elastic anisotropy in these crystals [46]. The sound velocities in the directions of *o*C12-AlAs, *h*P6-AlAs, and diamond-AlAs at different pressures are listed in Table 3. For orthorhombic symmetry, the sound velocities in the directions are obtained by the following expression [47]:

(9) $$\begin{array}{l} {}[100]:[100]v_{l}=\sqrt{C_{11}/\rho },\;[010]v_{t1}=\sqrt{C_{66}/\rho },\;[001]v_{t2}=\sqrt{C_{55}/\rho }\\ {}[010]:[010]v_{l}=\sqrt{C_{22}/\rho },\;[100]v_{t1}=\sqrt{C_{66}/\rho },\;[001]v_{t2}=\sqrt{C_{44}/\rho }\\ {}[001]:[001]v_{l}=\sqrt{C_{33}/\rho },\;[100]v_{t1}=\sqrt{C_{55}/\rho },\;[010]v_{t2}=\sqrt{C_{44}/\rho } \end{array}$$

For hexagonal symmetry, the sound velocities in the directions are obtained by the following expression [47]:

(10) $$\begin{array}{l} {}[100]:[100]v_{l}=\sqrt{(C_{11}-C_{12})/2\rho },\;[010]v_{t1}=\sqrt{C_{11}/\rho },\;[001]v_{t2}=\sqrt{C_{44}/\rho }\\ {}[001]:[001]v_{l}=\sqrt{C_{33}/\rho },\;[100]v_{t1}=[010]v_{t2}=\sqrt{C_{44}/\rho } \end{array}$$

For cubic symmetry, the sound velocities in the directions are obtained by the following expression [47]:

(11) $$\begin{array}{l} {}[100]:[100]v_{l}=\sqrt{C_{11}/\rho },\;[010]v_{t1}=[001]v_{t2}=\sqrt{C_{44}/\rho }\\ {}[110]:[110]v_{l}=\sqrt{(C_{11}+C_{12}+2C_{44})/2\rho },\;[1\bar{1}0]v_{t1}=\sqrt{(C_{11}-C_{12}）/\rho },\;[001]v_{t2}=\sqrt{C_{12}/\rho }\\ {}[111]:[111]v_{l}=\sqrt{(C_{11}+2C_{12}+4C_{44})/3\rho },\;[11\bar{2}]v_{t1}=[11\bar{2}]v_{t2}=\sqrt{(C_{11}-C_{12}+C_{44})/3\rho } \end{array}$$

where *ρ* is the density of AlAs; *v*_*l*_ is the longitudinal sound velocity; and *v_t_*_1_ and *v_t_*_2_ are the first transverse mode and the second transverse mode, respectively. As indicated by the above Equation (9), for orthorhombic symmetry, *C*_11_, *C*_22_, and *C*_33_ determine the longitudinal sound velocities along the [100], [010], and [001] directions, respectively, and *C*_44_, *C*_55_, and *C*_66_ correspond to the transverse sound velocities. For hexagonal symmetry, *C*_33_ determines the longitudinal sound velocity along the [001] direction, and *C*_11_ and *C*_44_ correspond to the transverse sound velocities (Equation (10)). It is obvious that the longitudinal sound velocities for *o*C12-AlAs along different directions increase with increasing pressure, and that the first and second transverse modes first increase and reach the maximum value at 6 GPa before decreasing. For *h*P6-AlAs, the longitudinal sound velocities changes in the [100] direction with no trend, whereas in the [001] direction, it first increases and reaches the maximum value at 8 GPa before decreasing. The changes of the first and second transverse sound velocities of *h*P6-AlAs also exhibit this initial increase and subsequent decrease regularity, but they reached their maximum values at different pressures. At 0 GPa, for *o*C12-AlAs (*h*P6-AlAs), the lowest sound velocity is 3151 m/s (3245 m/s), the highest sound velocity is 5999 m/s (5890 m/s), and the maximum (minimum) value of the sound velocity in the diamond phase is 6397 m/s (3305 m/s). In addition, at 10 GPa, the lowest sound velocity is 2711 m/s (3269 m/s), and the highest sound velocity is 6590 m/s (6225 m/s). The change trend is related to the corresponding elastic constants and indicates the elastic anisotropy of AlAs in different structures or under different pressures.

### 3.4. Thermal Properties

The sound velocity and Debye temperature (Θ*_D_*) are two fundamental parameters for evaluating the chemical bonding characteristics and thermal properties of materials in materials science. The Debye temperature is obtained by the following expressions [48,49]:

(12) $$\Theta_{D}=\frac{h}{k_{B}}{\left[\frac{3n}{4\pi }\left(\frac{N_{A}\rho }{M}\right)\right]}^{\frac{1}{3}}v_{m}$$

where *h* is Planck’s constant, *k_B_* is Boltzmann’s constant, *N_A_* is Avogadro’s number, *n* is the number of atoms in the molecule, *M* is the molecular weight, and *ρ* is the density. *v*_m_ is the average sound velocity, which can be obtained by the following expression [48]:

(13) $$v_{m}={\left[\frac{1}{3}(\frac{2}{v_{l}^{3}}+\frac{1}{v_{t}^{3}})\right]}^{-\frac{1}{3}}$$

where *v*_*l*_ and *v_t_* are the longitudinal and transverse sound velocities, respectively, which can be obtained from Navier’s equation [50]:

(14) $$v_{l}=\sqrt{(B+\frac{4}{3}G)\frac{1}{\rho }}$$

(15) $$v_{t}=\sqrt{\frac{G}{\rho }}$$

The density, sound velocity, and Debye temperature for *o*C12-AlAs and *h*P6-AlAs are listed in Table 4. The Debye temperature for diamond-AlAs in this work is in excellent agreement with the results of previous research. Usually for materials, the higher the Debye temperature, the greater the hardness. With the increase in pressure, the Debye temperature for *o*C12-AlAs and *h*P6-AlAs increases first and then decreases, with the maximum (484 K and 498 K, respectively) at 6 GPa. That is, at 6 GPa, the hardness becomes best, and the bonds become strongest. At ambient pressure, the Debye temperatures are 463 K, 471 K, and 433 K for *o*C12-AlAs, *h*P6-AlAs, and diamond-AlAs respectively, indicating that *o*C12-AlAs and *h*P6-AlAs have higher Debye temperature than diamond-AlAs. The longitudinal and transverse sound velocities of *o*C12-AlAs are similar to those of *h*P6-AlAs, because *o*C12-AlAs and *h*P6-AlAs have the similar elastic moduli. The density and *v*_*l*_ both increase with increasing pressure. With the increase in the pressure, the transverse sound velocities and average sound velocity show a non-monotonic increase or decrease. The average sound velocities of *h*P6-AlAs at different pressure are relatively large, exceeding 3600 m/s, which is slightly greater than those of *o*C12-AlAs (3400 m/s). In addition, at ambient pressure, the sound velocities are 3672 m/s, 3746 m/s, and 3562 m/s for *o*C12-AlAs, *h*P6-AlAs, and diamond-AlAs, respectively.

**Figure 5:**
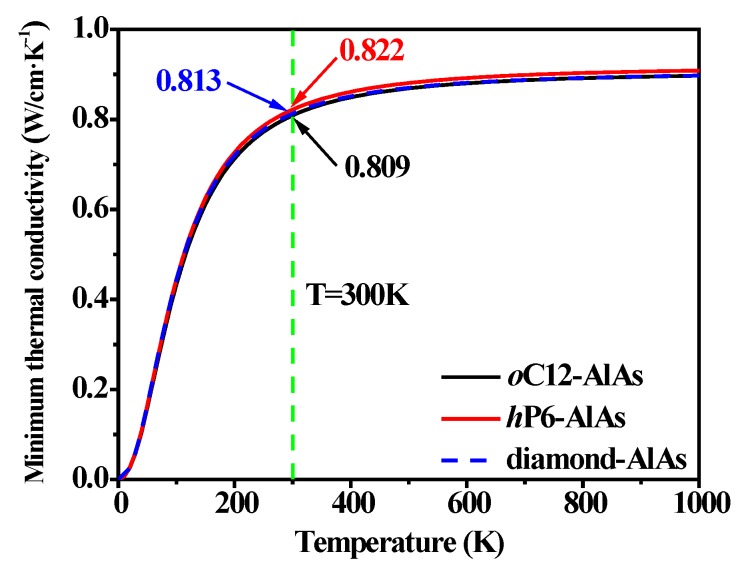
Temperature dependence of the minimum thermal conductivity for *o*C12-AlAs, *h*P6-AlAs, and diamond-AlAs at ambient pressure.

The thermal conductivity is the physical property of a material that conducts heat. Thermal conductivity can be used to determine the maximum power of a semiconductor device to operate and determine the efficiency of a semiconductor for thermoelectric energy conversion. The minimum thermal conductivity *κ*_min_ can be calculated by Cahill’s model theory, which is expressed as follows [52]:

(16) $$\kappa_{min}=\frac{\kappa_{B}}{2.48}N^{\frac{2}{3}}(v_{l}+2v_{t})$$

where *N* is the number of atoms in a conventional volume, and *v*_*l*_ and *v_t_* are the longitudinal and transverse sound velocities, respectively. To obtain the minimum thermal conductivity with the temperature changes, Cahill et al. found that *κ*_min_ as a function of temperature can be expressed by the following expression [51]:

(17) κmin=(π6)13kBn23Σivi(TΘi)2∫0ΘiTx3ex(ex−1)2dx

where *v_i_* represents the three acoustic modes (two transverse and one longitudinal). Θ*_i_* is the cutoff frequency for each polarization expressed in K, Θ*_i_* = *v_i_*[*h/*(2π*k*_B_)] (6π^2^*n*)^1/3^, and *n* is the number density of atoms. The temperature dependence of the minimum thermal conductivity for *o*C12-AlAs, *h*P6-AlAs, and diamond-AlAs at ambient pressure are shown in Figure 5. The thermal conductivities of *o*C12-AlAs, *h*P6-AlAs, and diamond-AlAs increase with the increase in temperature, and finally reach the corresponding stable value after 500 K. In the whole temperature range, the values of their minimum thermal conductivity *κ*_min_ are similar to each other. Among two AlAs isomers and diamond-AlAs, *h*P6-AlAs possesses the highest value, which is slightly higher than that of oC12-AlAs and diamond-AlAs, and the data curves of *o*C12-AlAs and diamond-AlAs almost coincide. At T = 300 K, the minimum thermal conductivity *κ*_min_ of *o*C12-AlAs, *h*P6-AlAs, and diamond-AlAs are 0.809 W/cm·K^−1^, 0.822 W/cm·K^−1^, and 0.813 W/cm·K^−1^, respectively. All of these investigation results illustrate that *h*P6-AlAs has the best thermal conductivity of the three structures, and *o*C12-AlAs is much close to diamond-AlAs.

## 4. Conclusions

In summary, the structural, mechanical, anisotropic, and thermal properties of *o*C12-AlAs, *h*P6-AlAs, and diamond-AlAs have been systematically investigated by employing first-principles calculations. The lattice constants ratios *a*/*a*_0_, *b*/*b*_0_, *c*/*c*_0_, and *V*/*V*_0_ show that the incompressibility of *o*C12-AlAs, and *h*P6-AlAs is largest along the *c*-axis, and the incompressibility of *o*C12-AlAs is slightly less than that of *h*P6-AlAs, but larger than diamond-AlAs. Also, the *C*_11_, *C*_22_, and *C*_33_ increase under increasing pressure, indicating that the greater the pressure, the more favorable the mechanical properties of *o*C12-AlAs and *h*P6-AlAs along the *a*, *b*, and *c* axes. At ambient pressure, the calculated bulk modulus and shear modulus have indicated that *o*C12-AlAs and *h*P6-AlAs have a better capability of resistance to volume and shape change than diamond-AlAs. According to the values of *B*/*G* and *v*, it is found that the pressures of transition from the brittle to ductile for *o*C12-AlAs, and *h*P6-AlAs are 1.21 GPa and 2.11 GPa, respectively. Detailed analyses of the anisotropy factors (Young’s modulus, Poisson’s ratio, and sound velocities, etc.) demonstrate that AlAs in diamond phase has the greatest anisotropy of the three phases. Furthermore, the Debye temperature and the minimum thermal conductivity at considered pressure were also investigated in this paper. At ambient pressure, the Debye temperature of *o*C12-AlAs, *h*P6-AlAs, and diamond-AlAs are 463 K, 471 K, and 433 K respectively. At *T* = 300 K, the minimum thermal conductivity *κ*_min_ of *o*C12-AlAs, *h*P6-AlAs, and diamond-AlAs are 0.809 W/cm·K^−1^, 0.822 W/cm·K^−1^, and 0.813 W/cm·K^−1^ respectively, indicating that AlAs in *o*C12 and *h*P6 phases have favorable thermal conductivity.

## Figures and Tables

**Table 1:** The lattice parameters *a*, *b*, *c* (in **Å**) of AlAs in the *o*C12, *h*P6, and diamond phases. PBE: Perdew–Burke–Ernzerhof.

Materials	PBE			Exp.
	*a*	*b*	*c*	*a*
diamond-AlAs	5.675			5.661 ^1^
*o*C12-AlAs	6.972	3.968	9.108	
	6.975 ^2^	3.977 ^2^	9.094 ^2^	
*h*P6-AlAs	4.019		8.990	
	4.026 ^2^		8.973 ^2^	

^1^ Ref [27], ^2^ Ref [35].

**Table 2:** The elastic constants (in GPa) and the elastic modulus (in GPa) of *o*C12-AlAs, *h*P6-AlAs, and diamond-AlAs under pressure.

Materials	P	*C*_11_	*C*_12_	*C*_13_	*C*_22_	*C*_23_	*C*_33_	*C*_44_	*C*_55_	*C*_66_	*B*	*G*	*E*	*v*
*o*C12-AlAs	0	120	41	50	130	45	145	40	52	42	76	44	111	0.257
	0 ^1^	127	39	45	121	52	153	47	38	43	74	43	108	0.257
	2	133	48	59	138	51	155	47	58	44	82	47	118	0.259
	4	137	56	65	145	59	163	44	55	41	89	46	118	0.280
	6	146	63	74	154	67	177	47	59	45	98	48	124	0.289
	8	156	68	82	161	73	186	33	41	45	105	42	111	0.324
	10	162	77	91	169	80	195	41	33	44	113	42	112	0.335
*h*P6-AlAs	0	127	42	49			140	53		43	75	46	115	0.245
	0 ^1^	126	38	51			147	44			75	44	110	0.256
	2	133	50	56			147	50		42	82	45	114	0.268
	4	140	57	64			158	52		41	90	46	118	0.282
	6	165	62	75			171	53		51	103	51	131	0.288
	8	165	67	82			182	53		49	108	50	130	0.299
	10	171	75	86			174	53		48	112	49	128	0.309
diamond-AlAs	0	116	53					55			74	44	110	0.252
	0 ^2^	120	57					57			78	45		

^1^ Ref [27], ^2^ Ref [36].

**Table 3:** The sound velocities along different directions of *o*C12-AlAs, *h*P6-AlAs, and diamond-AlAs under pressure.

Materials	Directions		Pressure					
			0	2	4	6	8	10
*o*C12-AlAs	[100]	[100]*v*_*l*_	5464	5675	5691	5813	5948	6007
		[010]*v*_*t*1_	3232	3264	3113	3227	3194	3130
		[001]*v*_*t*2_	3597	3747	3606	3696	3049	2711
	[010]	[010]*v*_*l*_	5687	5780	5855	5971	6042	6135
		[100]*v*_*t*1_	3232	3264	3113	3227	3194	3130
		[001]*v*_*t*2_	3154	3373	3225	3298	2736	3022
	[001]	[001]*v*_*l*_	6006	6126	6208	6401	6494	6590
		[100]*v*_*t*1_	3597	3747	3606	3696	3049	2711
		[010]*v*_*t*2_	3154	3373	3225	3298	2736	3022
*h*P6-AlAs	[100]	[100]*v*_*l*_	3243	3166	3129	3450	3333	3269
		[010]*v*_*t*1_	5607	5667	5747	6176	6116	6171
		[001]*v*_*t*2_	3622	3475	3502	3500	3466	3436
	[001]	[001]*v*_*l*_	5887	5958	6105	6287	6424	6225
		[100]*v*_*t*1_	3622	3475	3502	3500	3466	3436
		[010]*v*_*t*2_	3622	3475	3502	3500	3466	3436
diamond-AlAs	[100]	[100]*v*_*l*_	5676					
		[010]*v*_*t*1_	3909					
		[001]*v*_*t*2_	3909					
	[110]	[110]*v*_*l*_	6225					
		[1$\bar{1}$0]*v_t_*_1_	4183					
		[001]*v_t_*_2_	3837					
	[111]	[111]*v*_*l*_	6397					
		[11$\bar{2}$]*v_t_*_1_	3305					
		[11$\bar{2}$]*v_t_*_2_	3305					

**Table 4:** The density (*ρ* in g/cm^3^), sound velocity (*v*_*l*_, *v_t_*, *v_m_*, in m/s), and Debye temperature (Θ*_D_* in K) for *o*C12-AlAs, *h*P6-AlAs, and diamond-AlAs under pressure.

Materials	Pressure	*ρ* (g/cm^3^)	*v*_l_	*v*_t_	*v*_m_	Θ*_D_*
*o*C12-AlAs	0	4.02	5781	3305	3672	463
	2	4.13	5916	3372	3748	476
	4	4.23	5960	3297	3673	473
	6	4.32	6123	3333	3718	484
	8	4.41	6044	3087	3458	457
	10	4.49	6135	3058	3432	458
*h*P6-AlAs	0	4.04	5812	3376	3746	471
	2	4.14	5856	3297	3668	468
	4	4.24	5975	3294	3671	474
	6	4.33	6287	3433	3829	498
	8	4.41	6293	3367	3761	494
	10	4.49	6284	3303	3694	490
diamond-AlAs	0	3.60	6068	3495	3880	433
	0 ^1^	3.73				450

^1^ Ref [51].
